# ﻿A new genus of Textricini Lehtinen, 1967 (Araneae, Agelenidae) from Anatolia

**DOI:** 10.3897/zookeys.1151.100430

**Published:** 2023-02-28

**Authors:** Rahşen S. Kaya, Alireza Zamani, Ersen Aydın Yağmur, Yuri M. Marusik

**Affiliations:** 1 Department of Biology, Faculty of Arts and Science, Bursa Uludağ University, TR-16059, Bursa, Türkiye Bursa Uludağ University Bursa Turkiye; 2 Zoological Museum, Biodiversity Unit, FI-20014 University of Turku, Turku, Finland University of Turku Turku Finland; 3 Alaşehir Vocational School, Manisa Celal Bayar University, Manisa, Türkiye Manisa Celal Bayar University Manisa Turkiye; 4 Department of Zoology & Entomology, University of the Free State, Bloemfontein 9300, South Africa University of the Free State Bloemfontein South Africa

**Keywords:** Ageleninae, Aranei, dichotomous key, new species, Türkiye

## Abstract

Türkiye is known to have the highest diversity of the spider family Agelenidae in the Western Palaearctic and the highest diversity of the subfamily Ageleninae globally. The new agelenid genus *Anatextrix***gen. nov.** (Ageleninae, Textricini) and its type species, *A.spectabilis***sp. nov.** (♂♀; Mersin and Adana provinces, southern Türkiye), are described. A key to all four genera of Textricini is provided.

## ﻿Introduction

Agelenidae C.L. Koch, 1837 is a large family of spiders currently comprising 1374 extant species in 91 genera distributed worldwide (WSC 2023). According to Lehtinen (1967), two subfamilies – Ageleninae C.L. Koch, 1837 and Coelotinae F.O. Pickard-Cambridge, 1893 – can be recognized within Agelenidae, with the former comprising the following tribes: Agelenopsini Lehtinen, 1967 (Nearctic and Neotropical), Agelenini C.L. Koch, 1837 (Holarctic and Afrotropical), Tegenariini Lehtinen, 1967 (primarily Palaearctic), and Textricini Lehtinen, 1967 (Western Palaearctic). Textricini, the smallest tribe and the focus of this paper, is primarily distributed in the Mediterranean region (WSC 2023). Several new species of this tribe have been described over the past few years, including five from the Maghreb (Bosmans et al. 2022) and one from Anatolia (Dimitrov 2022).

Recently, we had the opportunity to examine specimens of a new species of Textricini from Türkiye. This species displays a series of interesting characters, including a strongly modified palpal femur bearing several processes or outgrowths, which is a unique trait in the whole family. Based on a comparison with other genera of Textricini, we decided that this undescribed species also represents a new genus. In this paper, both the new genus and species are described, and a key to all four genera of Textricini is provided.

## ﻿Materials and methods

The samples were collected with pitfall trapping and hand aspirator and preserved in 70% ethanol. Specimens were photographed using a Canon EOS 7D camera attached to an Olympus SZX16 stereomicroscope at the Zoological Museum of the University of Turku. Digital images were montaged using Combine ZP and edited using CorelDraw. Illustrations of internal genitalia were made after clearing and cleaning the epigyne in a 10% KOH aqueous solution, followed by a few minutes of treatment in Chlorazol Black. Lengths of leg segments were measured on the dorsal side and are listed as: total length (femur, patella, tibia, metatarsus, tarsus). All measurements are in millimetres (mm). Spination formula follows Bolzern et al. (2008, 2009).

### ﻿Abbreviations

Eyes: **ALE** ‒ anterior lateral eye, **AME** ‒ anterior median eye, **PLE** ‒ posterior lateral eye, **PME** ‒ posterior median eye.

Spination: **d** ‒ dorsal, **Fe** ‒ femur, **Mt** ‒ metatarsus, **Pa** ‒ patella, **pl** ‒ prolateral, **rl** ‒ retrolateral, **Ti** ‒ tibia, **v** ‒ ventral.

Male palp: **Bd** – dorsal branch of the conductor, **Ca** – anterior arm of the conductor, **Cf** – cymbial fold, **Cp** – posterior arm of the conductor, **Db** – distal bulge, **Eb** – base of the embolus, **Kt** – ventral keel, **Pb** – proximal bulge, **Pt** – prolateral apophysis, **Rt** – retrolateral apophysis, **So** – stump-like outgrowth, **Sp** – spine-like outgrowth, **St** – subtegulum, **Te** – tegulum, **Va** – ventral apophysis.

Epigyne: **Cd** – copulatory duct, **Fd** – fertilization duct, **Oc** – copulatory opening, **Re** – receptacle, **Se** – septum.

### ﻿Depositories

**AZMM** Alaşehir Zoological Museum of Manisa Celal Bayar University, Türkiye (E.A. Yağmur).

**ZMUT**Zoological Museum of the University of Turku, Finland (V. Vahtera).

**ZMUU** Zoological Museum of the Bursa Uludağ University, Türkiye (R.S. Kaya).

## ﻿Taxonomy

### ﻿Family Agelenidae C.L. Koch, 1837

**Subfamily Ageleninae C.L. Koch, 1837**

#### ﻿Tribe Textricini

Taxon classificationAnimaliaAraneaeAgelenidae

Lehtinen, 1967

61C7F5A6-E62E-5B85-9448-E467F2C24237

##### Diagnosis.

**Figure 1.**
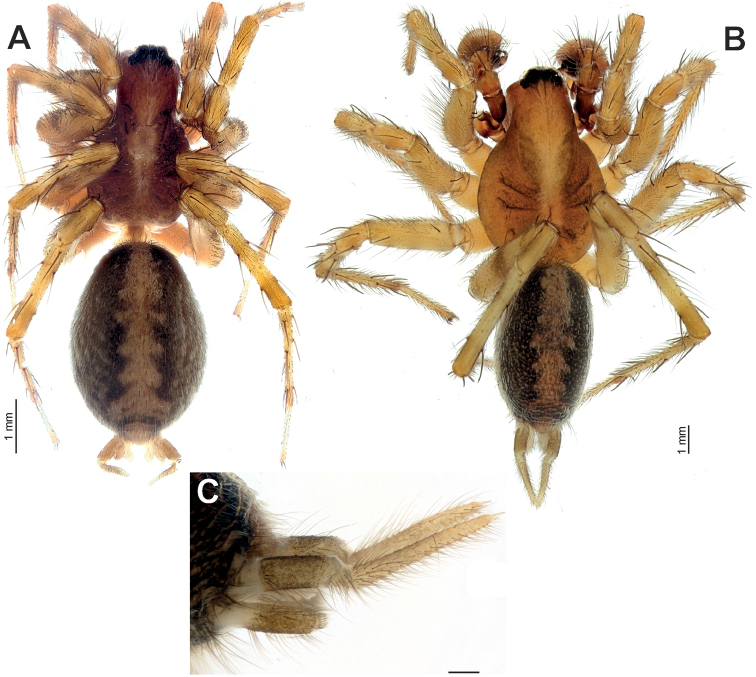
*Anatextrixspectabilis* sp. nov. **A** female habitus, dorsal view **B** male habitus, dorsal view **C** spinnerets of the male, lateral view. Scale bars: 0.2 mm, unless stated otherwise.

Species considered in Textricini have a very long terminal segment of the posterior lateral spinnerets (Fig. 1C), and a strongly recurved posterior eye row (vs procurved in Agelenini, straight in Tegenariini). Furthermore, males of Textricini species lack a tegular (= median) apophysis (vs present in all other agelenids; also see Discussion). For other characters, see Lehtinen (1967) and Bolzern et al. (2010, 2013).

##### Composition.

Thirty species in four genera: *Anatextrix* gen. nov. (one species), *Lycosoides* Lucas, 1846 (14 species), *Maimuna* Lehtinen, 1967 (eight species), and *Textrix* Sundevall, 1833 (seven species).

### ﻿Key to the genera

This key is primarily based on the characters of the generotypes: *Lycosoidescoarctata* (Dufour, 1831), *Maimunavestita* (C.L. Koch, 1841), and *Textrixdenticulata* (Olivier, 1789).

**Table:** 

1	Male	**2**
–	Female	**5**
2	Femur, patella and tibia with apophyses; tibia with prolateral apophysis; cymbium with prolateral fold (Figs 2A, 4A)	***Anatextrix* gen. nov.**
–	Femur without apophyses or only with minor modifications; prolateral tibial apophysis and prolateral cymbial fold are lacking	**3**
3	Palpal patella modified: swollen with one retrolateral apophysis (Fig. 8A, D)	***Lycosoides* Lucas, 1846**
–	Palpal patella not modified	**4**
4	Posterior arm of the conductor with two branches, one branch directed dorsally (*Bd*) and partly hiding cymbium; prolateral arm as large as tibia (Fig. 8B, E)	***Maimuna* Lehtinen, 1967**
–	Conductor different (Fig. 8C, F)	***Textrix* Sundevall, 1833**
5	Epigyne with scape and distinct, deep fovea	***Textrix***
–	Epigyne without scape and deep fovea	**6**
6	Epigyne with stripe-like septum	***Anatextrix* gen. nov.**
–	Epigyne without septum	**7**
7	Epigyne with anterior hood and anchor-like median plate; receptacles located meso-laterally	***Lycosoides***
–	Hood absent; receptacles located posteriorly and spaced by ca two diameters of each	***Maimuna***

#### ﻿Genus Anatextrix gen. nov.

Taxon classificationAnimaliaAraneaeAgelenidae

C5F01027-922D-55F8-BA34-BD01AC87D031

https://zoobank.org/3E23C193-9504-47BF-B38A-DDE6BAE44DEE

##### Type species.

*Anatextrixspectabilis* sp. nov.

##### Etymology.

The generic epithet is a combination of Anatolia and *Textrix*; gender feminine.

##### Diagnosis.

**Figure 2.**
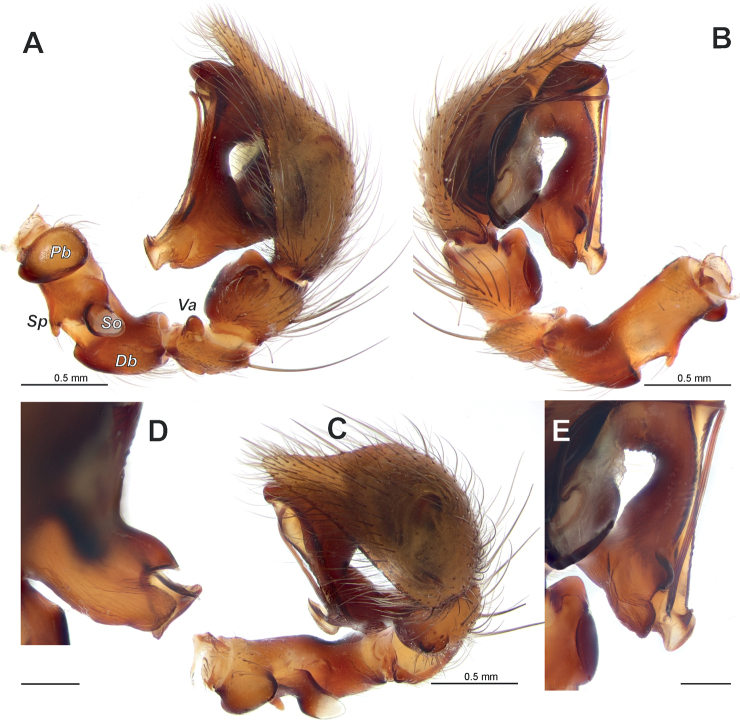
Male palp of *Anatextrixspectabilis* sp. nov. **A–C** full palp, retrolateral, prolateral and retrodorsal views **D, E** details of the embolus and the posterior arm of the conductor, prodorsal and prolateral views. Scale bars: 0.2 mm, unless stated otherwise. Abbreviations: *Db* – distal bulge, *Pb* – proximal bulge, *So* – stump-like outgrowth, *Sp* – spine-like outgrowth, *Va* – ventral apophysis.

The new genus differs from all other genera of Textricini by having a strongly modified male palpal femur with two outgrowths and two bulges (vs one or none), presence of the palpal prolateral tibial apophysis (*Pt*) and the cymbial prolateral fold (*Cf*) (vs lacking), the straight mesal part of the embolic base (vs round) (cf. Figs 2A, 4A, 8A–F), and by having a thin septum in the epigyne (vs absent). Furthermore, the female of *Anatextrixspectabilis* sp. nov. differs from those of *Textrix* by having no epigynal fovea and scape (vs present). From the females of the two other genera, the female of this species differs by the anterior position of the receptacles (vs mesal or posterior).

##### Description.

Same as for the type species.

##### Composition.

Only the type species.

##### Distribution.

Same as for the type species.

#### ﻿ Anatextrix spectabilis sp. nov.

Taxon classificationAnimaliaAraneaeAgelenidae

44382823-923E-56F6-9CE2-090F37CBBA91

https://zoobank.org/A95490E1-44D5-411A-BFF0-17B6E3F877AD

**Figure 3.**
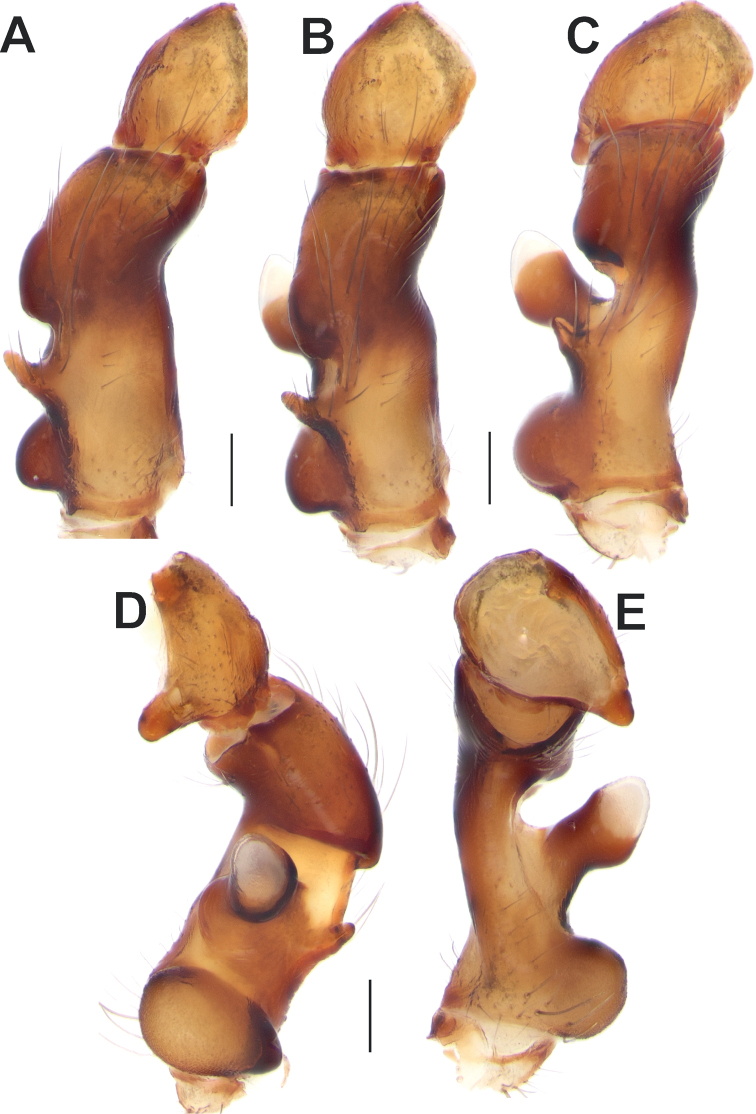
Male palpal femur and patella of *Anatextrixspectabilis* sp. nov. **A** prolateral view **B** prodorsal view **C** dorsal view **D** retrolateral view **E** ventral view. Scale bars: 0.2 mm.

**Figure 4.**
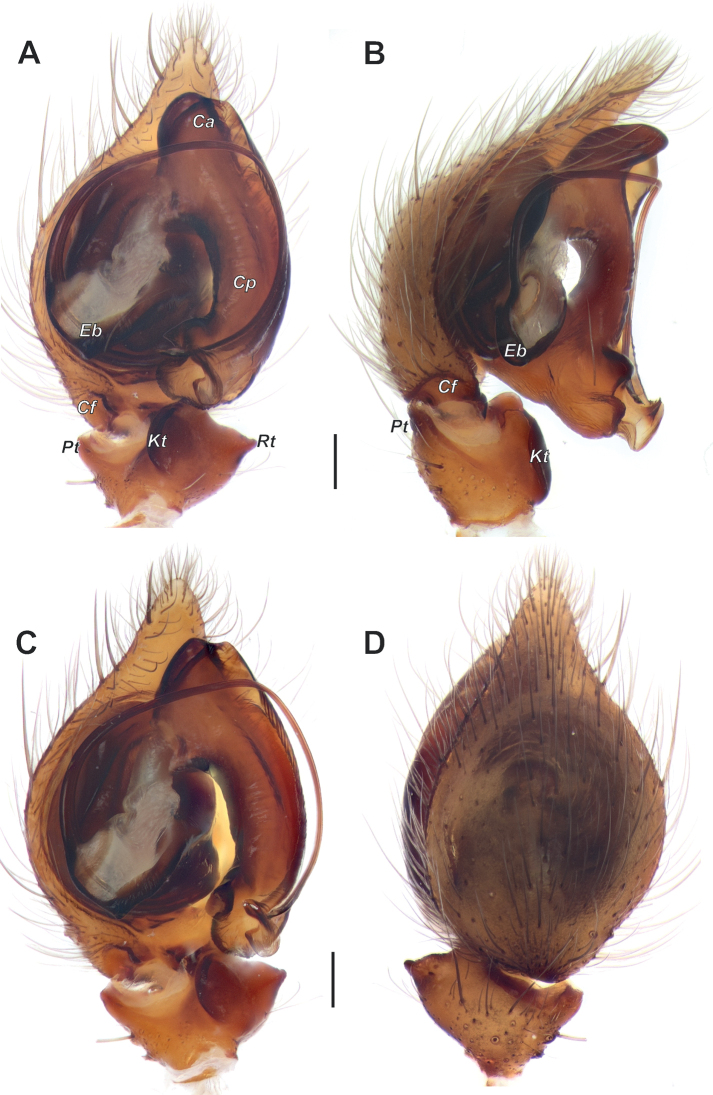
Male palp of *Anatextrixspectabilis* sp. nov. **A** ventral view **B** prolateral view **C** proventral view **D** dorsal view. Scale bars: 0.2 mm. Abbreviations: *Ca* – anterior arm of the conductor, *Cp* – posterior arm of the conductor, *Cf* – cymbial fold, *Eb* – base of the embolus, *Kt* – ventral keel, *Pt* – prolateral apophysis, *Rt* – retrolateral apophysis.

**Figure 5.**
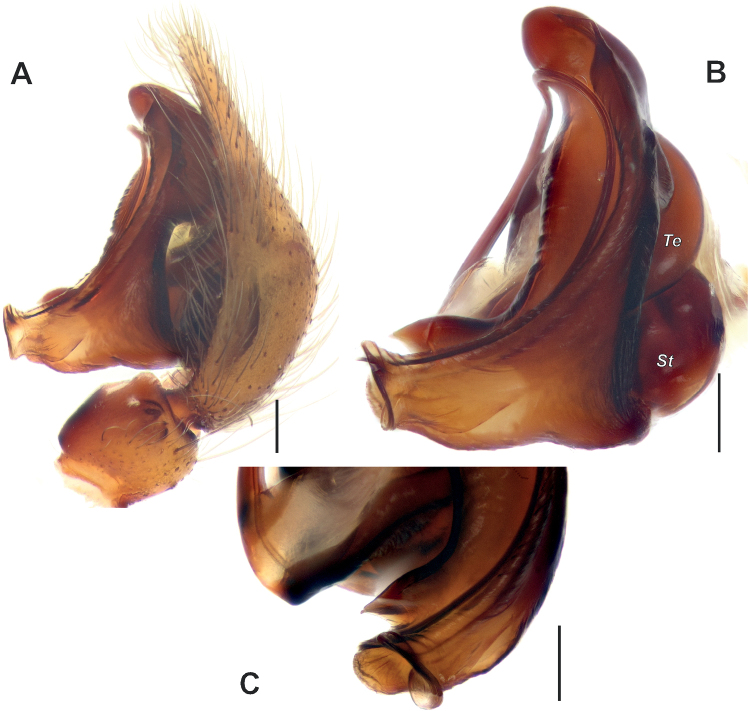
Male palp of *Anatextrixspectabilis* sp. nov. **A** retrolateral view **B** dissected bulb, retrolateral view **C** details of the embolus and the posterior arm of the conductor, anteroventral view. Scale bars: 0.2 mm. Abbreviations: *St* – subtegulum, *Te* – tegulum.

**Figure 6.**
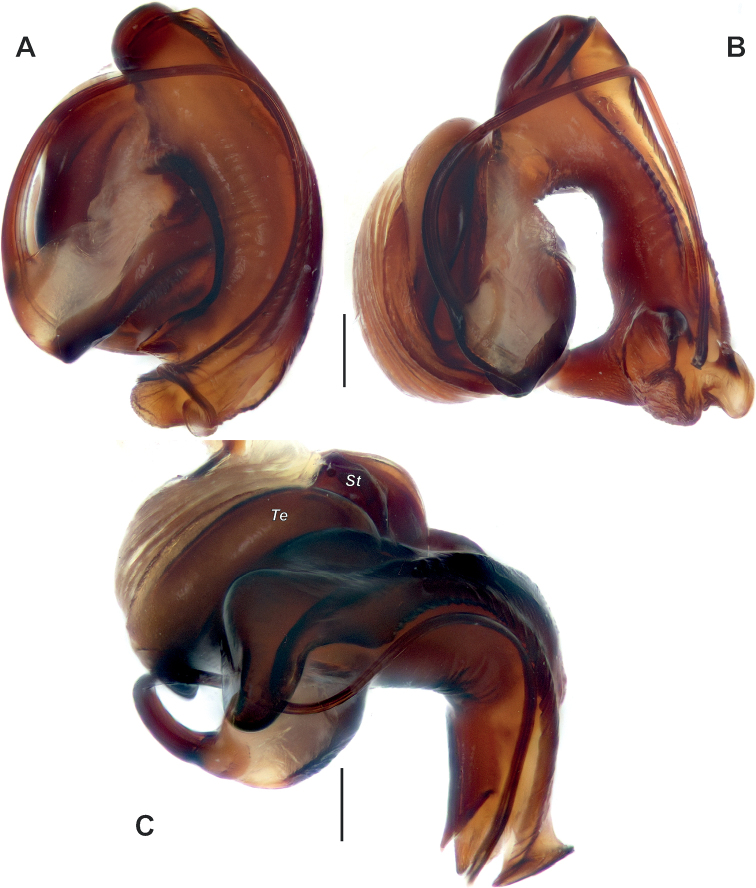
Dissected bulb of *Anatextrixspectabilis* sp. nov. **A** ventral view **B** prolateral view **C** anterior view. Scale bars: 0.2 mm. Abbreviations: *St* – subtegulum, *Te* – tegulum.

**Figure 7.**
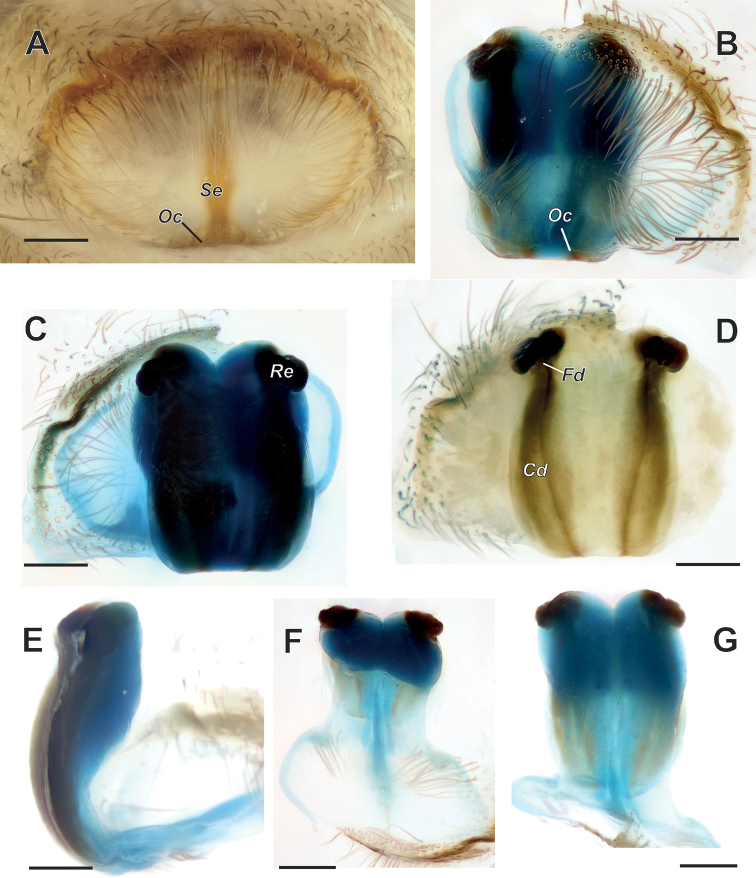
Epigyne of *Anatextrixspectabilis* sp. nov. **A** intact, ventral view **B, G** macerated, ventral view **C, D** vulva, dorsal view **E, F** same, lateral and anterior views. Scale bars: 0.2 mm. Abbreviations: *Cd* – copulatory duct, *Fd* – fertilization duct, *Oc* – copulatory opening, *Re* – receptacle, *Se* – septum.

Figs 1A–C , 2A–E , 3A–E , 4A–D , 5A–C , 6A–C , 7A–G

##### Type material.

***Holotype*** ♂ (ZMUU), Türkiye: **Mersin Province**: Erdemli district, 36°44'N, 34°09'E, 960 m a.s.l., 18.07.2015, hand collection (E.A. Yağmur). ***Paratypes***: 1♂2♀ (ZMUT), same data as for the holotype; 1♂17♀ (ZMUU), same data as for the holotype; **Adana Province**: 2♂12♀ (ZMUU), Pozantı district, 37°25'58"N, 34°55'11"E, 1396 m a.s.l., 27.09.2018, hand collection (R.S. Kaya and E.A. Yağmur); 2♂2♀ (AZMM), same locality, 31.10.2017–02.04.2018, pitfall traps, (E.A. Yağmur); 8♂3♀ (ZMUU), same locality, 31.10.2017–02.04.2018, pitfall traps, (E.A. Yağmur); 15♂6♀ (ZMUU), same locality, 04.08.2018–19.07.2019, pitfall traps, (E.A. Yağmur).

##### Etymology.

The specific epithet is a Latin adjective meaning “remarkable”.

##### Diagnosis.

Same as for the genus.

##### Description.

**Male (Holotype).** Habitus as in Fig. 1B. Total length 6.55. Carapace 3.57 long, 1.22 wide at pars cephalica, 2.37 wide at pars thoracica. Eye sizes: AME: 0.11, ALE: 0.15, PME: 0.22, PLE: 0.16. Carapace, sternum, labium, and maxillae light brown; carapace with darker submedian bands. Chelicerae light reddish brown, each with 3 pro- and 2 retromarginal teeth. Legs yellowish brown, with annulations. Abdomen dorsally dark greyish with lighter foliate pattern, light greyish ventrally. Spinnerets light greyish, darker basally (Fig. 1C). Measurements of legs: I: 7.48 (2.00, 0.90, 1.53, 1.95, 1.10), II: 7.75 (2.15, 0.89, 1.53, 2.00, 1.18), III: 7.78 (2.00, 0.93, 1.60, 2.20, 1.05), IV: 9.64 (2.55, 0.84, 1.95, 2.97, 1.33). Spination is given in Table 1.

**Table 1.** Spination of legs of *Anatextrixspectabilis* sp. nov. The letter “p” indicates paired spines.

		Fe	Pa	Ti	Mt
		d-pl-rl	d-pl-rl	d-pl-rl-v	pl-rl-v
**I**	♂	3-1-1	2-1-1	1-2-1-1+2p	1-1-3p
	♀	3-1-1	2-1-1	1-2-1-1+1p	2-2-2p
**II**	♂	3-1-1	2-1-1	1-2-1-1+1p	2-2-3p
	♀	3-1-1	2-1-1	1-2-1-1+1p	2-2-3p
**III**	♂	3-1-1	2-1-1	1-2-2-1+1p	3-2-3p
	♀	3-1-1	2-1-1	1-2-3-3p	3-3-3p
**IV**	♂	3-1-1	2-1-1	2-2-2-2+1p	3-3-3p
	♀	3-1-1	2-1-1	2-2-2-2+1p	3-3-3p

Palp as in Figs 2A–E, 3A–E, 4A–D, 5A–C, 6A–C; femur relatively short (ca 3× longer than wide, 1.5× shorter than cymbium) and strongly modified: slightly bent, with proximal (*Pb*) and distal (*Db*) bulges and 2 outgrowths: spine-like (*Sp*) and larger stump-like (*So*). Patella short, wider than long, with ventral apophysis (*Va*). Tibia slightly wider than long in retrolateral view, with retrolateral (*Rt*) conical apophysis directed laterally, ventral keel (*Kt*) and prolateral apophysis (*Pt*). Cymbium droplet-shaped, ca 1.7× longer than wide, with small baso-prolateral fold (*Cf*). Subtegulum (*St*) round, hidden by tegulum and conductor. Tegulum (*Te*) small, round, hidden by conductor and embolus base. Conductor very large, ca 0.7× shorter than cymbium; anterior and posterior parts extending over tegulum; anterior arm (*Ca*) as long as wide, posterior arm (*Cp*) more than 2× longer than wide; posterior part of posterior arm very broad and extending ventrally; tip of posterior arm trifurcate. Embolus proper originates at about 8 o’clock position and terminates at about 5 o’clock position; base of embolus (*Eb*) not rounded: mesal part straight, prolateral part bent on right angle.

**Female (Paratype, ZMUT).** Habitus as in Fig. 1A. Total length 6.75. Carapace 2.95 long, 1.16 wide at pars cephalica, 2.97 wide at pars thoracica. Eye sizes: AME: 0.10, ALE: 0.15, PME: 0.22, PLE: 0.14. Coloration generally as in male, except for darker submedian bands on carapace and more distinct annulations on legs. Measurements of legs: I: 6.55 (1.80, 0.87, 1.30, 1.58, 1.00), II: 6.73 (1.87, 0.93, 1.30, 1.57, 1.06), III: 6.98 (1.90, 0.90, 1.33, 1.85, 1.00), IV: 9.05 (2.29, 1.04, 1.92, 2.60, 1.20). Spination is given in Table 1.

Epigyne as in Fig. 7A–G; epigynal plate 1.6× wider than long; fovea lacking depression, weakly sclerotized with thin septum (*Se*), anterior part delimited by well sclerotized margin, posterior part delimited by weakly sclerotized margins; septum not covered by setae; copulatory openings (*Oc*) located on posterior margin on both sides of septum; copulatory ducts (*Cd*) fused, forming a rectangular plate ca 2× longer than wide (Fig. 7G); receptacles (*Re*) small, suboval, located anteriorly and separated by ca one length of each; fertilization ducts (*Fd*) short and small.

##### Natural history.

The specimens were collected off their funnel-webs constructed under the stones or within shrubs and crevices in the soil. The habitat is a typical maquis shrubland dominated by *Quercus* L. (Fagaceae) and *Pinus* L. (Pinaceae) at Erdemli (Mersin), while it is dominated by *Abies* Mill. (Pinaceae) at Pozantı (Adana).

##### Distribution.

Known only from the provinces of Mersin and Adana, southern Türkiye.

## ﻿Discussion

In this paper, a new genus and species of Textricini are described from southern Türkiye. Since many species of Textricini have characteristics that differ from the type species of the genera in which they are currently classified, this tribe, as a whole, needs to be revised. Furthermore, both *Lycosoides* and *Textrix* comprise species that show considerable differences in the conformation of their copulatory organs, and, thus, might be misclassified. This is most likely why de Blauwe (1980) considered all Textricini species known at that time to belong to *Textrix*.

**Figure 8.**
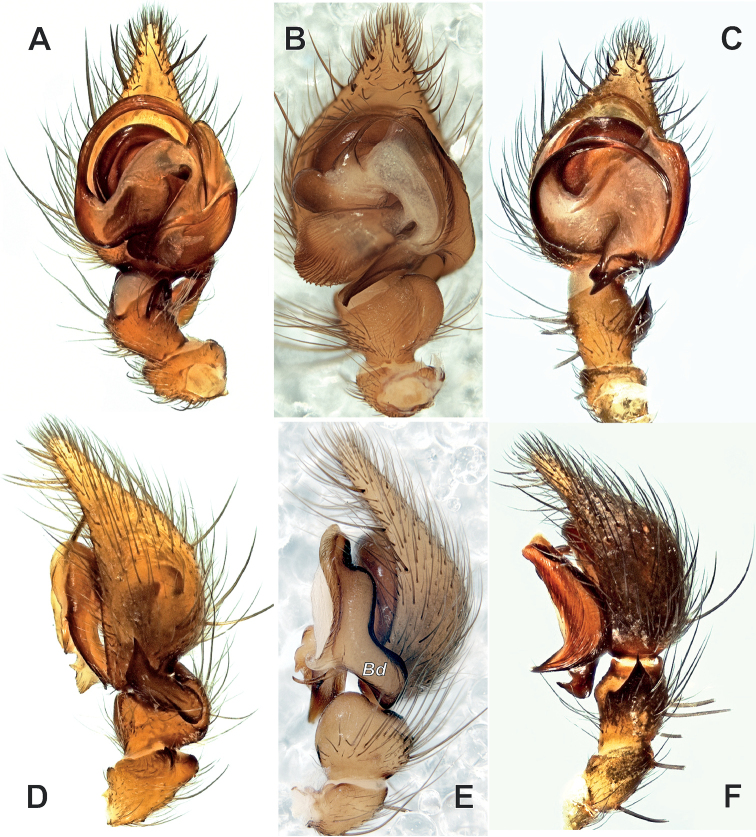
Male palps of the type species of three genera belonging to Textricini: *Lycosoidescoarctata* (**A, D**), *Maimunavestita* (**B, E**) and *Textrixdenticulata* (**C, F**) **A–C** ventral view **D–F** retrolateral view **A, C, D, F** courtesy of P. Oger **B, E** from Dimitrov (2022), courtesy of D. Dimitrov. Abbreviation: *Bd* – dorsal branch of the conductor.

The terminology of one particular palpal sclerite in *Maimuna* has been controversial: Lehtinen (1967) suggested that a tegular (= median) apophysis is lacking (i.e., “totally reduced”) in species of this genus, which was not followed by Levy (1996), Bolzern et al. (2013), and Dimitrov (2022). It became evident in our examination of the expanded bulbs that all species of *Maimuna* indeed lack a tegular apophysis, as the structure that the aforementioned authors considered as the “median apophysis” arises from the conductor, not the tegulum.

Agelenidae is relatively well studied in Türkiye due to several regional revisions and other taxonomic and faunistic contributions (e.g., Brignoli 1972, 1978a, b; Kaya et al. 2010; Danışman and Karanfil 2015; Danışman et al. 2016; Topçu and Demircan 2018; Dimitrov 2022). Currently, there are 72 species in 16 genera of Agelenidae known from Türkiye (Danışman et al. 2022; present paper), which is considerably higher than what is known from, for example, Iran (25 species in seven genera; Zamani and Marusik 2019, 2020), the entire Caucasus (36 species in 11 genera; Otto 2022), Greece (48 species in 12 genera; Nentwig et al. 2023), Bulgaria (43 species in 11 genera; Nentwig et al. 2023), Italy (58 species in 14 genera; Nentwig et al. 2023), France (41 species in 13 genera; Nentwig et al. 2023), and Spain (41 species in 13 genera; Nentwig et al. 2023). Indeed, in terms of the diversity of agelenids in the Palaearctic, Türkiye is only second to China (>445 species in 35 genera; Li 2020). It is noteworthy that Türkiye houses the highest number of Ageleninae species globally.

Although most of the Turkish agelenids belong to Tegenariini (including 19 endemic species), Textricini is also relatively diverse in this country (i.e., eight species in all four known genera, including one endemic genus and two endemic species; Danışman et al. 2022; present paper). As it has been mentioned earlier, members of this tribe are primarily distributed in the Mediterranean region. The only exceptions are *Lycosoideslehtineni* Marusik & Guseinov, 2003 from Azerbaijan and *Textrixnigromarginata* Strand, 1906 from Ethiopia, although both are known only from females and the latter is most likely misclassified (Strand 1906, 1908; Marusik and Guseinov 2003).

Despite the relatively well-explored status of the Turkish agelenids, new species and records are still found regularly. Most likely there are many interesting species of Agelenidae in this country that are currently undiscovered, as it is evidenced by the remarkable new genus described in this paper. Hopefully, a more complete picture of the diversity of this family in Türkiye can be achieved once the lesser explored habitats (e.g., caves) and regions (e.g., eastern Türkiye) are systematically surveyed.

## Supplementary Material

XML Treatment for Textricini

XML Treatment for Anatextrix

XML Treatment for Anatextrix spectabilis
